# Efficacy of Statin Treatment in Early-Stage Chronic Kidney Disease

**DOI:** 10.1371/journal.pone.0170017

**Published:** 2017-01-12

**Authors:** Eun Yeong Cho, Chana Myoung, Hong-suk Park, Ae Jin Kim, Han Ro, Jae Hyun Chang, Hyun Hee Lee, Wookyung Chung, Ji Yong Jung

**Affiliations:** 1 Division of Nephrology, Department of Internal Medicine, Gachon University of Gil Medical Center, Incheon, Republic of Korea; 2 Division of Nephrology, Department of Internal Medicine Gachon University School of Medicine, Incheon, Republic of Korea; Hospital Universitario de la Princesa, SPAIN

## Abstract

Chronic kidney disease (CKD) represents a major medical challenge and frequently coexists with cardiovascular disease (CVD), which can be treated by statin trerapy. However, whether statin treatment affects renal progression and outcomes in CKD patients remains unclear. We retrospectively reviewed CKD patients at Gachon University Gil Medical Center from 2003–2013. From a total of 14,497 CKD patients, 858 statin users were paired with non-users and analyze with propensity score matching was performed. The outcomes of this study were creatinine doubling, renal death, all-cause mortality, and interactive factors for composite outcomes. Statins were prescribed to 13.5% of the study subjects. Hazard ratios (HRs) [95% confidence intervals (CIs)] for statin treatment for the doubling of serum creatinine levels were significant only in CKD patients with an estimated glomerular filtration rate (eGFR) ≥30 mL/min/1.73 m^2^, and were 0.744 (0.635–0.873) in the unmatched cohort and 0.767 (0.596–0.986) in the matched cohort. In analyses of secondary outcomes, the HRs (95% CIs) for all-cause mortality were 0.655 (0.502–0.855) in the unmatched cohort and 0.537 (0.297–0.973) in the matched cohort. The HRs (95% CIs) for statin therapy for composite outcomes among patients with and without an eGFR ≥30 mL/min/1.73 m^2^ were 0.764 (0.613–0.952) and 1.232 (0.894–1.697), respectively (*P* for interaction, 0.017). Thus, statin treatment may have beneficial effects on renal progression and all-cause mortality only for the patients with early- stage CKD.

## Introduction

Chronic kidney disease (CKD) has been emerged as an important health concern and is a major cause of premature mortality and comorbidities [1]. Individuals with CKD have a higher risk of premature mortality related to cardiovascular disease (CVD) even during the early stages of disease; this represents a significant challenge for physicians [2]. Renal dysfunction increases inflammation, oxidative stress, platelet dysfunction, endothelial dysfunction, proteinuria, and electrolyte imbalances [3–6]. Moreover, these alterations may be related to a higher risk of CVD events [7]. Dyslipidemia and hypertension are common conditions associated with CVD [7–9]. Various toxic lipid intermediates are formed in dyslipidemic CKD patients, which may accelerate the progression of CKD [10–12]. Therefore, optimal strategies for the management of dyslipidemia are needed to prevent CVD, particularly in CKD patients.

In previous studies, statin therapy improved renal function and reduced proteinuria [13–15], possibly due to their anti-inflammatory activity and improvement of endothelial function, which reduces abnormal permeability to plasma proteins [6, 16–20]. Thus, statin administration may benefit CKD patients due to its enhancement of renal perfusion by improving endothelial and cardiac function [20, 21]. However, the indications for statin treatment are unclear, and whether it slows the progression of CKD and improves renal outcomes is controversial [22, 23]. The latest guidelines for lipid management in CKD patients from Kidney Disease: Improving Global Outcomes (KDIGO) do not suggest an appropriate point for the initiation of statin therapy; indeed, whether statin therapy has beneficial effects in all CKD patients is also uncertain [22, 23]. Therefore, we evaluated the clinical impact of statin treatment on the clinical outcomes of patients with CKD according to disease stage.

## Materials and Methods

### Study design and participants

**Fig 1:**
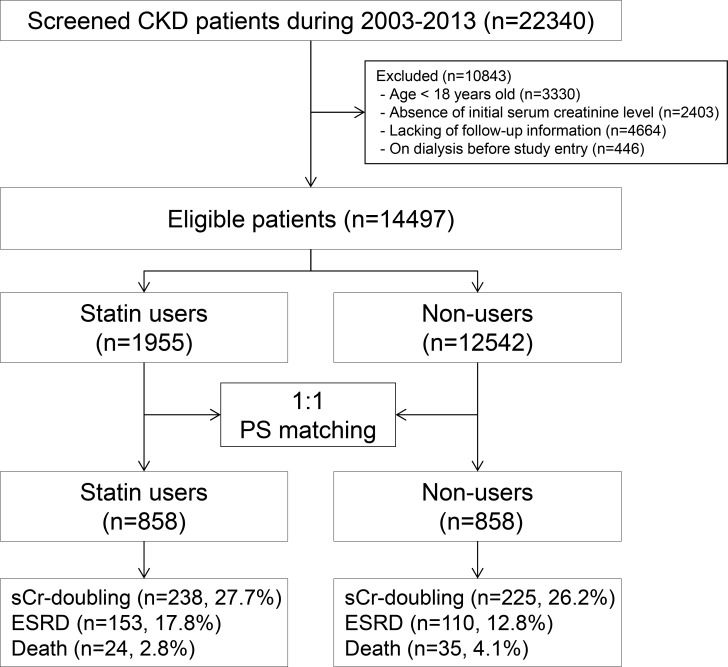
Cohort formation.

We performed a propensity score (PS)-matched analysis of a retrospective observational cohort. Data from a total of 22,340 patients with CKD between January 1, 2003, and August 31, 2013, were included. Of these patients, an analysis of 14,497 patients was performed after excluding the following groups: (i) those less than 18 years of age (n = 3,330); (ii) patients with missing information concerning comparable data (n = 2,403) or follow up (n = 4,664); and (iii) those who had previously undergone dialysis or transplantation before the index date (start point of observation, baseline creatinine measurement; n = 446; Fig 1). The patients were divided into two cohorts based on their statin prescription. Statin users (n = 1,955) were prescribed statins at least 3 months before the index date. Non-users (n = 12,542) were not prescribed statins before the index date. The research protocol and methods were approved by the Institutional Review Board (IRB) of Gachon University Gil Medical Center (GAIRB2016-085). The IRB waived the requirement for written informed consent as this was a retrospective study that did not include any interventions.

### Variables

Demographic (age, sex, and body mass index [BMI]), clinical (medication information and medical history of diabetes, hypertension and previous CVD), and laboratory (serum levels of creatinine, hemoglobin, white blood cells, platelet, albumin, calcium, phosphorus, uric acid, cholesterol, triglycerides, high-density lipoprotein [HDL]-cholesterol, and the presence of proteinuria) data of patients were obtained from electronic medical records. CVD was defined as major vascular diseases such as myocardial infarction and other ischemic heart diseases, including angina pectoris and heart failure, atrial fibrillation, and cerebrovascular disease. The four-variable Modification of Diet in Renal Disease (MDRD) Study equation was used to calculate the estimated glomerular filtration rate (eGFR) [24]. We defined and staged the CKD according to the eGFR (<60 mL/min/1.73 m^2^), proteinuria manifestation, or both, on two occasions at least 3 months apart [25].

### Outcomes

Primary outcomes were doubling of serum creatinine and renal events reaching the end-stage renal disease (ESRD) necessitating renal replacement therapy. Secondary outcomes were all-cause mortality and composite outcomes (creatinine doubling, renal death, and all-cause mortality). Outcomes were compared on the index date until the first event.

### Statistical analysis

To decrease any potential confounding bias, PS matching was adopted to adjust for significant differences in baseline covariates. As described previously [26], the probability for statin prescription was determined using a multiple logistic regression analysis model (C statistic = 0.624; Hosmer and Lemeshow goodness-of-fit test, P = 0.1494), which included demographics and clinical and laboratory values (age, sex, BMI, medical history of diabetes, hypertension, and previous CVD; baseline eGFR; presence of proteinuria, hemoglobin, albumin, calcium, phosphorus, and uric acid; cholesterol, triglycerides, and HDL-cholesterol levels; and the prescription of RAAS blockers, beta blockers, CCBs, diuretics, and aspirin). After PS matching, the balance in baseline covariates between the two groups was assessed using the standardized mean difference test, paired t-test, and McNemar’s test. PS matching was constructed using the Statistical Analysis Systems (SAS) software package version 9.3 (SAS Institute, Cary, NC).

A Cox proportional regression was performed to determine the adjusted hazard ratios (HRs) for statin prescription with 95% confidence intervals (CIs) for outcomes. Stratified subgroup analyses to test for confounding variables and interactions were also performed. All data analyses with the exception of PS matching were performed using Statistical Package for the Social Sciences (SPSS) software version 20.0 (IBM Corp., Armonk, NY). A value of *P* < 0.05 was considered to indicate statistical significance.

## Results

### Patient characteristics

A total of 14,497 CKD patients met the inclusion criteria and were followed up for a median of 30 months (interquartile range: 3–67 months). Statins were prescribed to 13.5% (1,955 of 14,497) of the patients. In particular, stain use was more common in older and male patients. Moreover, medical comorbidities and the prescription of other medications were showed more frequent in the statin user group than in non-users. After PS matching of 858 statin users with non-users, no significant differences in baseline characteristics were found between the two groups. (Table 1).

**Table 1:** Baseline characteristics of study participants (before after PS 1:1 matching).

	Before matching				After PS matching			
Variables	Statin users (N = 1955)	Non-users (N = 12542)	*P*	Standardized differences	Statin users (N = 858)	Non-users (N = 858)	*P*	Standardized differences
Age, year	55.40±13.48	48.92±15.91	**<0.001**	0.603	53.86±13.41	53.37±15.31	0.483	0.059
Male gender, n (%)	1036 (53.0)	6313 (50.3)	**0.029**	0.054	457 (53.3)	482 (56.2)	0.233	0.058
Diabetes, n (%)	791 (40.5)	1475 (11.8)	**<0.001**	0.691	289 (33.7)	260 (30.3)	0.137	0.073
Hypertension, n (%)	1380 (70.6)	2946 (23.5)	**<0.001**	1.070	635 (74.0)	649 (75.6)	0.420	0.037
BMI, kg/m^2^	25.90±5.76	25.49±6.45	0.076	0.075	26.90±6.74	27.69±7.51	0.290	0.053
Previous CVD, n (%)	460 (23.5)	683 (5.4)	**<0.001**	0.533	141 (16.4)	124 (14.5)	0.271	0.037
eGFR, ml/min/1.73m^2^	56.88±27.98	69.57±29.70	**<0.001**	0.615	57.82±30.06	59.01±33.46	0.434	0.056
Proteinuria, n (%)	1172 (59.9)	5984 (47.7)	**<0.001**	0.247	532 (62.0)	547 (63.8)	0.640	0.061
Laboratory								
Hemoglobin, g/dl	13.16±2.28	13.35±2.13	**0.002**	0.062	13.00±2.33	13.07±2.25	0.615	0.018
White blood cells, x10^3^/ml	7.95±2.89	7.78±3.77	**0.041**	0.059	7.86±2.80	7.94±3.09	0.631	0.075
Platelet, x10^3^/ml	266.70±78.78	258.90±77.59	**<0.001**	0.137	265.80±78.24	262.80±75.98	0.505	0.035
Albumin, g/dl	4.04±0.66	4.25±0.51	**<0.001**	0.131	4.06±0.62	4.05±0.64	0.769	0.005
Cholesterol, mg/dl	221.43±65.21	183.68±43.43	**<0.001**	0.802	212.21±64.55	208.20±55.15	0.154	0.102
Triglycerides, mg/dl	200.93±171.02	140.10±105.89	**<0.001**	0.502	180.84±133.26	186.95±172.38	0.419	0.037
HDL-cholesterol, mg/dl	48.72±15.04	50.54±14.63	**<0.001**	0.155	48.87±15.21	49.55±15.34	0.385	0.049
Calcium, mg/dl	9.01±0.73	9.01±0.62	0.889	0.001	8.95±0.72	8.96±0.70	0.835	0.007
Phosphorus, mg/dl	3.74±0.89	3.65±1.86	**0.038**	0.063	3.74±0.93	3.69±0.89	0.297	0.028
Uric acid, mg/dl	6.27±2.14	5.61±2.00	**<0.001**	0.293	6.37±2.17	6.40±2.16	0.813	0.028
Statins								
Simvastatin	654 (33.5)				235 (27.4)			
Rosuvastatin	459 (23.5)				217 (25.3)			
Pravastatin	394 (20.2)				199 (23.2)			
Atorvastatin	302 (15.4)				144 (16.8)			
Pitavastatin	95 (4.9)				48 (5.6)			
Lovastatin	51 (2.6)				15 (1.7)			
Other medications								
RAAS blockers, n (%)	1308 (66.9)	2183 (17.4)	**<0.001**	1.158	617 (71.9)	640 (74.6)	0.135	0.061
Beta-blockers, n (%)	956 (48.9)	1630 (13.0)	**<0.001**	0.843	432 (50.3)	396 (46.2)	0.074	0.082
CCB, n (%)	1085 (55.5)	1894 (15.1)	**<0.001**	0.933	454 (52.9)	475 (55.4)	0.306	0.050
Diuretics, n (%)	1048 (53.6)	1815 (14.5)	**<0.001**	0.906	444 (51.7)	439 (51.2)	0.831	0.010
Aspirin, n (%)	540 (27.6)	807 (6.4)	**<0.001**	0.588	202 (23.5)	175 (20.4)	0.118	0.075

BMI: body mass index; CVD: cardiovascular disease; eGFR: estimated glomerular filtration rate; HDL: high-density lipoprotein; RAAS: renin-angiotensin-aldosterone system; CCB: calcium channel blocker. *Note*: Conversion factors for units were as follows: hemoglobin in g/dl to g/l, × 10; albumin in mg/dl to g/l, × 10; cholesterol mg/dl to mmol/l, × 0.02586: triglycerides mg/dl to mmol/l, × 0.01129; HDL-cholesterol mg/dl to mmol/l, × 0.02586; LDL-cholesterol mg/dl to mmol/l, × 0.0258

### Primary outcome

Multivariable Cox proportional regression analyses were performed for doubling of serum creatinine or renal events (long-term dialysis and kidney transplantation) according to the stages of CKD (eGFR ≥30 mL/min/1.73 m^2^ or <30 mL/min/1.73 m^2^). Statins delayed the doubling of serum creatinine without considering eGFR in the unmatched cohort (HR, 0.824; 95% CI, 0.722–0.939; *P* = 0.004; Table 2), but this significant association was not seen in the matched cohort (HR, 0.871; 95% CI, 0.711–1.066; *P* = 0.181; Table 2). For the eGFR ≥30 mL/min/1.73 m^2^ group, statins significantly reduced the incidence of creatinine doubling in both the unmatched (HR, 0.744; 95% CI, 0.635–0.873; *P* < 0.001; Table 2) and matched (HR, 0.767; 95% CI, 0.596–0.986; *P* = 0.039; Table 2) cohorts. There was no significant relationship between statin treatment and creatinine doubling in the eGFR <30 mL/min/1.73 m^2^ group. In addition, the risk of renal events was not related to statin use in the unmatched (HR, 1.041; 95% CI, 0.866–1.250; *P* = 0.671) and matched (HR, 1.095; 95% CI, 0.833–1.440; *P* = 0.515; Table 2) cohorts.

**Table 2:** Association between statin and risk of CKD progression and risk of ESRD.

	Unmatched cohort		Matched cohort	
	HR (95% CI)	*P*	HR (95% CI)	*P*
SCr x 2^a^				
Non-users	1 (reference)		1 (reference)	
Statin users	0.824 (0.722–0.939)	**0.004**	0.871 (0.711–1.066)	0.181
eGFR≥30ml/min/1.73m^2^	0.744 (0.635–0.873)	**<0.001**	0.767 (0.596–0.986)	**0.039**
eGFR<30ml/min/1.73m^2^	0.992 (0.786–1.253)	0.948	1.101 (0.773–1.569)	0.595
Simvastatin	0.742 (0.618–0.891)	**0.001**	0.689 (0.518–0.918)	**0.011**
Rosuvastatin	1.009 (0.909–1.120)	0.868	1.076 (0.923–1.254)	0.349
Pravastatin	1.017 (0.944–1.096)	0.656	1.034 (0.931–1.150)	0.532
Atorvastatin	0.999 (0.940–1.062)	0.999	1.004 (0.922–1.093)	0.936
Pitavastatin	0.772 (0.648–0.921)	**0.004**	0.824 (0.655–1.036)	0.098
Lovastatin	0.991 (0.840–1.169)	0.916	1.043 (0.749–1.452)	0.804
ESRD				
Non-users	1 (reference)		1 (reference)	
Statin users	1.040 (0.866–1.249)	0.675	1.095 (0.833–1.440)	0.515
eGFR≥30ml/min/1.73m^2^	0.884 (0.673–1.161)	0.374	1.142 (0.753–1.733)	0.532
eGFR<30ml/min/1.73m^2^	1.220 (0.953–1.563)	0.115	1.136 (0.784–1.646)	0.500
Simvastatin	0.970 (0.769–1.225)	0.800	0.875 (0.619–1.238)	0.451
Rosuvastatin	1.093 (0.948–1.260)	0.220	1.130 (0.924–1.383)	0.233
Pravastatin	1.081 (0.977–1.197)	0.131	1.136 (0.999–1.293)	0.053
Atorvastatin	0.956 (0.873–1.048)	0.341	0.940 (0.829–1.065)	0.330
Pitavastatin	0.831 (0.662–1.044)	0.111	0.946 (0.749–1.195)	0.641
Lovastatin	0.991 (0.840–1.169)	0.916	1.043 (0.749–1.452)	0.804

Abbreviations: HR, hazards ratio; CI, confidence interval; SCr, serum creatinine; ESRD, end-stage renal disease. HRs were obtained from Cox models adjusted for age (years), diabetes, hypertension, previous CVD, proteinuria, baseline eGFR, hemoglobin level (< 10 g/dl), albumin level (< 3.5 g/dl), and cholesterol level and use of medications (RAAS blockers, aspirin, beta-blockers, CCB, and diuretics)

^a^SCr x 2: a doubling of the baseline serum creatinine concentration

### All-cause mortality

Statin users showed a lower mortality rate than those of non-users before (*P* = 0.002) and after PS matching (*P* = 0.04; Table 3). In a multivariable Cox proportional analysis, statin users had a lower incidence of mortality than non-users in both the unmatched cohort (HR, 0.655; 95% CI, 0.502–0.855; *P* = 0.002; Table 3) and in the matched cohort (HR, 0.537; 95% CI, 0.297–0.973; *P* = 0.040; Table 3). In the eGFR ≥30 mL/min/1.73 m^2^ group, statin users showed significantly less mortality risk in both the unmatched (HR, 0.518; 95% CI, 0.365–0.734; *P* < 0.001; Table 3) and matched (HR, 0.457; 95% CI, 0.214–0.979; *P* = 0.044; Table 3) cohorts. However, statin treatment did not significantly affect all-cause mortality in the patients with an eGFR <30 mL/min/1.73 m^2^.

**Table 3:** Association between statin and risk for all-cause mortality.

	Unmatched cohort		Matched cohort	
	HR (95% CI)	*P*	HR (95% CI)	*P*
All-cause mortality				
Non-users	1 (reference)		1 (reference)	
Statin users	0.655 (0.502–0.855)	**0.002**	0.537 (0.297–0.973)	**0.040**
eGFR≥30ml/min/1.73m^2^	0.518 (0.365–0.734)	**<0.001**	0.457 (0.214–0.979)	**0.044**
eGFR<30ml/min/1.73m^2^	0.946 (0.619–1.446)	0.798	0.752 (0.248–2.279)	0.752
Simvastatin	0.640 (0.435–0.942)	**0.024**	0.450 (0.172–1.178)	0.104
Rosuvastatin	1.021 (0.827–1.260)	0.845	0.917 (0.570–1.475)	0.721
Pravastatin	0.892 (0.741–1.075)	0.231	0.850 (0.571–1.264)	0.421
Atorvastatin	0.906 (0.784–1.048)	0.185	1.002 (0.791–1.269)	0.987
Pitavastatin	0.923 (0.734–1.159)	0.489	0.985 (0.656–1.479)	0.940
Lovastatin	0.969 (0.768–1.223)	0.790	—	—

Abbreviations: HR, hazards ratio; CI, confidence interval; SCr, serum creatinine. HRs were obtained from Cox models adjusted for age (years), diabetes, hypertension, previous CVD, proteinuria, baseline eGFR, hemoglobin level (< 10 g/dl), albumin level (< 3.5 g/dl), and cholesterol level and use of medications (RAAS blockers, aspirin, beta-blockers, CCB, and diuretics)

### Other secondary endpoints; associations of statin use with composite outcomes according to eGFR

**Fig 2:**
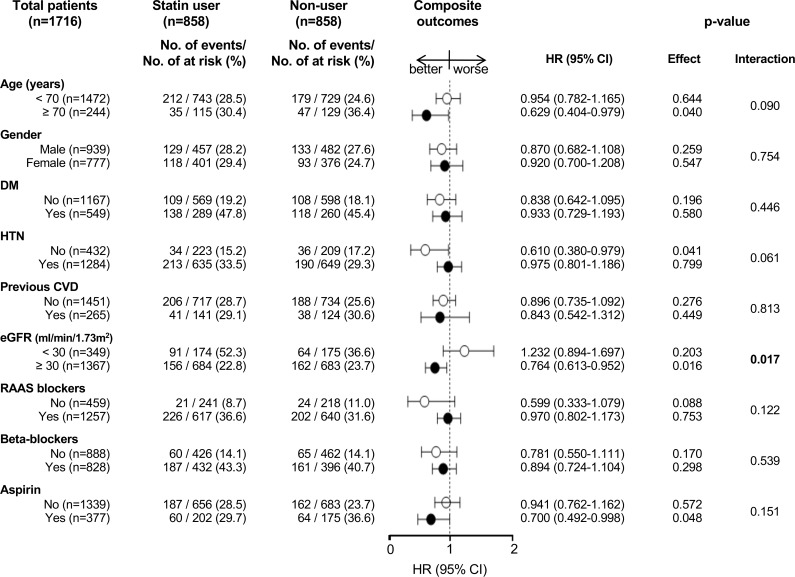
Association of statin treatment and composite outcomes in subgroups of matched cohort.

In the matched cohort, 79.6% (1,367 of 1,716) of the patients had early-stage CKD (stages 1 to 3, eGFR ≥30 mL/min/1.73 m^2^). Among statin users in the PS-matched cohort, 22.8% (156 of 684) of patients with early-stage CKD were statin users, while 52.3% (91 of 174) of late-stage CKD patients (stages 4 and 5; eGFR <30 mL/min/1.73 m^2^) were statin users. The HRs (95% CIs) for composite outcomes among early- and late-stage CKD patients were 0.764 (0.613–0.952, *P* = 0.016) and 1.232 (0.894–1.697, *P* = 0.203), respectively (*P* interaction, 0.017; Fig 2). The influence of statin treatment on outcome was consistent across the stratified patient subgroups (Fig 2).

## Discussion

The effect of statin prescription on clinical outcomes in patients with CKD according to stage was evaluated using PS matching. We found that statin treatment delayed the deterioration of kidney function and reduced all-cause mortality only in patients with early-stage CKD (eGFR ≥30 mL/min/1.73 m^2^). Thus, we may consider statin treatment in such patients.

Several studies have evaluated the mechanism by which statins affect CKD [3, 12–15, 21, 27, 28]. Statins not only lower lipid profiles, they also exert anti-proteinuric effects [13]. However, the beneficial effects of statins had not previously been assessed according stage of CKD, dosage, and treatment duration. A previous study compared the renoprotective effects of atorvastatin and rosuvastatin in progressive renal disease patients with or without diabetes (PLANET I and PLANET II) [27]. The results suggested rosuvastatin to have a greater lipid-lowering effect. In contrast, atorvastatin exerts a greater renoprotective effect in CKD patients. Therefore, the renoprotective effect varies according to the type of statin; moreover, there was no correlation between the reduction in lipid levels and renoprotection.

Statins’ renoprotective effects may be dosage-dependent. High-dose statin therapy ameliorated the decline in eGFR in CKD patients (eGFR <60 mL/min/1.73 m^2^), while statin therapy had no effect on renal progression in patietns with low-dose prescriptions [3]. Hence, the renoprotective effect of statins may vary according to the dose and type of statin, as well as CKD stage. Although the effects of statins in patients with CKD of various stages are uncertatin, a favorable effect of statins has empirically been adopted in these populations; indeed, statins are frequently used in CKD patients. In our study, simvastatin exerted beneficial effects on renal progression and all-cause mortality only for patients with early-stage CKD. However, there were no significant differences on outcomes regarding outcomes when different doses of simvastatin were compared (data not shown).

The first guidelines for lipid therapy in CKD patients were published in 2003. According to those guidelines, treatment should be considered for CKD patients with a low-density lipoprotein (LDL) level >100 mg/dL to reduce the LDL level to <100 mg/dL [29]. As indicated above, statins are recommended in patients with all stages of CKD. However, two large randomized trials failed to show a beneficial effect of statin treatment in patients undergoing hemodialysis [30, 31]. The Die Deutsche Diabetes Dialysis (4D) trial [30] and The Assessment of Survival and Cardiovascular Events (AURORA) trial [31] demonstrated that statin therapy in dialysis patients lowers the LDL level but does not affect the incidence of CVD or all-cause mortality. Therefore, the revised guidelines suggested that statin treatment should not be initiated in patients on maintenance hemodialysis [32]. Subsequently, the authors reported a clear benefit of statins, even in patients undergoing hemodialysis in the Study of Heart and Renal Protection (SHARP) [33]. However, the efficacy of statins in CKD patients undergoing hemolysis have remains uncertain. The 2014 KDIGO guidelines recommend not initiating statin therapy in adults with dialysis-dependent CKD and continuation in adults taking statins at the time of dialysis initiation [22]. KDIGO guidelines on lipid management adopt a risk-based approach for statin therapy in accordance with ACC-AHA guidelines [23]. However, the guidelines did not suggest the time at which statin treatment should be commenced. Our findings suggest that statin therapy in early-stage CKD patients may increase the time until serum creatinine doubles and decrease all-cause mortality.

In the subgroup analysis, only those with early-stage CKD had better outcomes when taking statins. We evaluated the association between statin use and eGFR. The use of statins by patients whose disease has advanced to ESRD requiring renal replacement therapy is controversial, and results regarding statin use in dialysis patients are inconsistent [30, 31, 33]. It would be worth performing randomized controlled interventional studies to confirm the clinical usefulness of statins in terms of clinical outcome in dialysis patients. However, statins may benefit patients not undergoing dialysis. In agreement with these observations, our findings suggest that statins may exert an eGFR-dependent renoprotective effect.

Although our findings show that statin use is associated with delayed progression of CKD after adjustment for underlying CKD, this study had several limitations. First, we could not conclude whether efficacy depends on statin type or dosage. To address this, we need to determine whether high-dose statin treatment lengthens the doubling time for serum creatinine and whether it decreases the risk of ESRD and mortality in a future study considering the dosage and type of statin therapy. Another limitation is the involvement of a single center and an ethnically homogenous population. An additional, multiple-center study of statin efficacy in CKD patients of other ethnicities is thus warranted. Also, this was a retrospective observational study, therefore is not a perfect substitute for a randomized trial. Even though PS matched analyses were performed, out study may contain biased results due to differences in unmeasured characteristics. Despite these limitations, this was a large-scale observational cohort study that used PS matching to enhance the statistical significance of the findings.

In summary, the lipid-lowering effect of statins prevents cardiovascular events and may exert similar effects in CKD patients. Little is known about the effect of statins in ESRD patients or the point at which statin treatment should be initiated in CKD patients. This study demonstrates that statins improved the prognosis of early-stage CKD patients with an eGFR ≥ 30 mL/min/1.73 m^2^, as evidenced by a reduced time to serum creatinine doubling and decreased all-cause mortality.

In conclusion, although the effect of statins on renal progression in CKD patients is unclear, our results suggest statins to have beneficial effects in patients with an eGFR ≥30 mL/min/1.73 m^2^. Therefore, the commencement of statin treatment during the early stages of CKD may be beneficial. Future randomized controlled studies are warranted to confirm the efficacy of statin therapy in early-stage CKD patients.
